# On-Line Control of Feast/Famine Cycles to Improve PHB Accumulation during Cultivation of Mixed Microbial Cultures in Sequential Batch Reactors

**DOI:** 10.3390/ijerph182312611

**Published:** 2021-11-30

**Authors:** Francisco Cabrera, Álvaro Torres-Aravena, Fernanda Pinto-Ibieta, José Luis Campos, David Jeison

**Affiliations:** 1 Instituto de Ciencias Químicas Aplicadas, Universidad Autónoma de Chile, Avenida Alemania 01090, Temuco 4810101, Chile; f.cabrera01@ufromail.cl; 2 Department of Chemical Engineering, Universidad de La Frontera, Av. Francisco Salazar 01145, Temuco 4811230, Chile; f.pinto@proyectos.uct.cl; 3 Escuela de Ingeniería Bioquímica, Pontificia Universidad Católica de Valparaíso, Av. Brasil 2085, Valparaíso 2362803, Chile; alvaro.torres@pucv.cl; 4 Departamento de Procesos Industriales, Facultad de Ingeniería, Universidad Católica de Temuco, Avenida Rudecindo Ortega 02950, Temuco 4781312, Chile; 5 Facultad de Ingeniería y Ciencias, Universidad Adolfo Ibáñez, Avda. Padre Hurtado 750, Viña del Mar 2562340, Chile; jluis.campos@uai.cl

**Keywords:** PHB, bioplastics, SBR, dissolved oxygen, feast, famine

## Abstract

Production of polyhydroxyalkanoates (PHA) has generated great interest as building blocks for bioplastic production. Their production using mixed microbial cultures represents an interesting alternative, since it enables the use of organic wastes as a carbon source. Feast/famine strategy is a common way to promote selection of microorganisms with PHA accumulation capacity. However, when using waste sources, changes in substrate concentration are expected, that may affect performance and efficiency of the process. This study showed how the dissolved oxygen level can be used for online control of the cycle time, ensuring that the desired feast/famine ratio is effectively applied. An operation strategy is presented and validated, using sequential batch reactors fed with acetate as the carbon source. Production of polyhydroxybutyrate (PHB) was studied, which is the expected type of PHA to be synthetized when using acetate as substrate. Two reactors were operated by applying the proposed control strategy, to provide F/F ratios of 0.2 and 0.6, respectively. A third reactor was operated with a fixed cycle time, for comparison purposes. Results showed that the reactor that operated at an F/F ratio of 0.6 promoted higher biomass productivity and PHB content, as a result of a better use of available time, preventing unnecessary long famine times. The application of the tested strategy is a simple a reliable way to promote a better performance of feast/famine-based bioreactors involving mixed microbial cultures for PHB production.

## 1. Introduction

The increasing use of plastics in packing and other applications has triggered its accumulation in lands and waters, generating diverse ecological and environmental problems [1]. This has created increasing interest in producing biodegradable polymers suitable for the manufacture of thermoplastic materials, as an alternative to petroleum-based plastics. Polyhydroxyalkanoate (PHAs) are certainly among them. 

PHAs are bacterial polymers destined for energy storage. They are only produced by microorganisms that possess the enzymatic pool to transform carbon to polyester chains and store them in intracytoplasmic granules [2]. Traditionally, the industrial production of PHAs has been linked to the selection of microbial strains that allocate a large extent of their cellular content into storage [3]. As a result, high biomass PHA contents have been reported [4,5,6]. Nevertheless, these PHA production routes have turned out to be, so far, more expensive than petroleum-based polyesters production, because of the costs associated with substrate, culture conditions and PHA extraction [7]. PHA production using mixed microbial cultures (MMCs) has been proposed as an alternative way to produce PHA, and reports dealing with this alternative have steadily increased in the past two decades. MMCs have the versatility that comes from the operation under non-axenic environments, so no sterilization process is needed. Moreover, MMCs tend to be more robust to changes in operating conditions, which makes them a suitable alternative for large-scale production of PHAs [8,9]. The production of PHAs using MMCs normally involves an operation with consecutive periods of availability and scarcity of substrate, using sequential batch reactors (SBR). This strategy, normally called feast/famine (F/F), induces the selection of microorganisms that accumulate PHAs [10].

The cost of the substrate is one of the most relevant factors determining economic feasibility of PHA production [11,12]. Therefore, the use of substrates derived from solid wastes and wastewaters seem to be a suitable way to decrease production costs [13,14,15]. Moreover, such a strategy would increase the sustainability of produced PHAs, by the transformation of waste into valuable compounds. However, the use of waste-derived substrates imposes a major challenge, considering that wastes can be variable in terms of flow, concentration and composition. 

Organic load variability could be problematic for PHA production using F/F strategy. Operation cycles of SBRs are traditionally programmed using fixed cycle time intervals [16,17]. Then, variation of operational conditions will produce modifications of the relation between feast and famine periods. For example, an increase in organic load will induce a longer feast, producing a shorter famine, when total cycle time is kept constant. Previous reports have shown that changes in operational conditions, such as organic load rate and cycle time, can greatly affect performance of SBRs for PHA production [11,18]. Then, it seems clear that application of different organic loads would require adjustments of feast and famine times.

This study presents and evaluates a strategy for the selection of PHA-accumulating biomass, when system is subjected to periodic changes in organic load. The strategy involves the online adjustment of SBR operation cycles, in order to provide a constant F/F ratio, despite changes in applied organic load. It is based on the monitoring of dissolved oxygen (DO), as an input to calculate the necessary time of famine period, to keep a constant F/F ratio. The strategy was tested on SBR reactors, and results were contrasted with data from an SBR operated with fixed cycle time.

## 2. Materials and Methods

### 2.1. Inoculum and Feed Composition for SBR Reactors

Three SBR reactors of 2 L useful volume were operated to conduct this study. Reactors were implemented using modified glass bottles, provided with mechanical mixing and aeration. All SBRs were fed with the same synthetic media, containing sodium acetate as carbon source. The medium was also composed of 600 mg L^−1^ MgSO_4_·7H_2_O, 160 mg L^−1^ NH_4_Cl, 100 mg L^−1^ EDTA, 92 mg L^−1^ K_2_HPO_4_, 45 mg L^−1^ KH_2_PO_4_, 70 mg L^−1^ CaCl_2_·2H_2_O, 10 mg L^−1^ thiourea and 2 mL L^−1^ of trace element solution. The trace solution consisted of 1500 mg L^−1^ FeCl_3_·6H_2_O, 150 mg L^−1^ H_3_BO_3_, 150 mg L^−1^CoCl_2_·6H_2_O, 120 mg L^−1^ MnCl_2_·4H_2_O, 120 mg L^−1^ ZnSO_4_·7H_2_O, 60 mg L^−1^ Na_2_MoO_4_·2H_2_O, 30 mg L^−1^ CuSO_4_·5H_2_O and 30 mg L^−1^ of KI. Since acetate was used as carbon source, polyhydroxybutyrate (PHB) will be the produced PHA [9]. Consequently, PHB was the polymer measured in the biomass. 

Reactors were inoculated with biomass that was previously cultivated during 30 days in a supplementary SBR, using a cycle time of 6 h and 60 mM acetate as substrate. This supplementary SBR was in turn inoculated with aerobic sludge coming from the sewage treatment plant of Temuco city, Chile. Biomass cultivation in this supplementary SBR was carried out in order to obtain an inoculum already containing a large fraction of PHB producing microorganisms.

### 2.2. Operation of SBRs with Fixed and Non Fixed F/F Ratios

Operation cycles of SBRs included feeding, aerobic reaction phase and mixed liquor withdrawal. In all cases feeding time was 6 min, during which 250 mL of media were fed. Withdrawal of 250 mL was performed by the end of each cycle. No settling was applied, so hydraulic and biomass retention times were the same. Aeration and mechanical mixing were supplied during the whole operation, at 4 L min^−1^ and 60 rpm, respectively.

Peristaltic pumps (Masterflex-Cole Parmer, Vernon Hills, IL, USA) were used for liquid feed and withdrawal. Dissolved oxygen was acquired online by means of an optic industrial probe (WQ401, Global Water, College Station, TX, USA). Signals from sensors and pumps control were handled using a CompactDAQ system (cDAQ-9178 chassis, National Instruments, Austin, TX, USA), connected to a PC running a routine specially programmed for this purpose using LabView software (National Instruments, Austin, TX, USA).

Two influent acetate concentrations were used: 30 and 120 mM, which were applied alternately: during one cycle the reactor was fed with 30 mM acetate, during the next one, with 120 mM acetate, and so on. The alternation on influent concentration was achieved by preparing two feeds, and by using a control valve to automatically switch the substrate injection from one to the other. Since fed volume per cycle was constant, this produced changes on the applied organic load on each cycle. This operation strategy was selected, since it provides the desired dynamic conditions for reactor feeding, and can be easily implemented.

Two reactors were operated with the proposed strategy, which automatically adjusted cycle time, to provide F/F ratios of 0.2 (reactor R0.2) and 0.6 (reactor R0.6). The F/F ratio of 0.2 was considered based on previous studies dealing with PHA accumulation by biomass [17,19,20]. The F/F ratio of 0.6 was considered as a contrast condition, since it showed high PHB accumulation levels on author’s previous study [18]. A third reactor was operated as a control (reactor RC), at a constant total cycle time of 12 h. Table 1 describes the main operational conditions of each reactor.

Reactors were monitored for a period of time long enough to provide constant biomass concentration and stable F/F dynamics of PHB production and degradation during each cycle.

### 2.3. DO-Based F/F Ratio Control for PHB-Accumulating SBRs

The F/F boundary was determined based on the fast DO concentration increase that characterizes the end of the feast phase. Then, by determining the moment when DO exceeds a previously determined threshold concentration, the length of feast could be determined online, by a simple routine. In the case of this research threshold, DO concentration was fixed at 5 mg L^−1^, based on the observation of DO evolution during reactors operation.

### 2.4. Batch Experiments for PHB Production 

Once a steady operation was achieved, a sample of biomass from each reactor was extracted and mixed with culture medium, in order to perform batch PHB accumulation experiments. 125 mL of mixed liquor was mixed with a 30 mM acetate solution in a 1:1 volumetric proportion (250 mL final volume). Then, samples were incubated in an orbital shaker, at 150 rpm and 25 °C until all acetate was depleted from the medium. The mineral composition of the media was the same than that used for reactor operation, but removing the main nitrogen source (NH_4_Cl) to enhance the PHB accumulation through nitrogen depravation. Batch tests were evaluated in terms of PHB and biomass production.

### 2.5. Analytical Methods

Acetate concentration was measured on filtered samples (0.45 μm pore size) by gas-chromatography (Nukol™ capillary column 25 m, 0.25 mm), using a flame ionization detector (FID) (Clarus 400, Perkin Elmer, Waltham, MA, USA). Biomass was determined as volatile suspended solids (VSS), determined according to Standard Methods [21].

PHB content of biomass was determined according to Ivanova et al. [22]. A 5 mL sample was collected from the culture. Five drops of formaldehyde were added, and then samples were freeze-dried. Biomass was re-suspended in 1 mL acidic methanol (20% H_2_SO_4_) with 0.65 mg mL^−1^ benzoic acid as internal standard. Molecular sieves (0.3 nm) were added for water adsorption. Hydroxybutyric acid was measured then by gas chromatography (FID detector). A calibration curve was done using a standard of hydroxybutyric acid sodium salt. 

### 2.6. Calculations

Cycle times, productivities, PHB production and other parameters that characterize the operation of the reactors were based on data from three consecutive cycles, after a stationary phase was achieved. PHB productivity was calculated as the overall mass of PHB leaving the reactor during each cycle, divided by reactor volume and cycle time. Initial and maximum PHB concentrations refer to the values observed at the beginning of the cycle and at the end of feast phase, respectively.

## 3. Results

### 3.1. SBRs Overall Performances 

**Figure 1:**
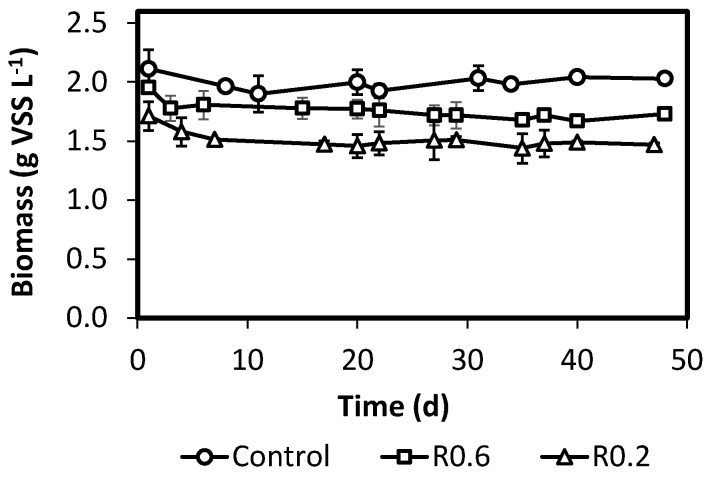
Biomass content of different SBRs during the operation time.

Figure 1 presents the biomass concentration during the operation of reactors R0.6, R0.2 and RC. Since biomass used for inoculation of the SBRs was obtained from an already F/F managed system, small changes in biomass concentration were observed during operational period. R0.6 and R0.2 showed an average biomass content of 1.8 and 1.5 g VSS L^−1^ respectively, while RC showed a slightly higher value, 2.0 g VSS L^−1^. In general, SBRs showed stable biomass concentrations and F/F ratios from third week until the end of the operation. Therefore, kinetic parameters of PHB production were evaluated after one month of continuous stable operation. 

**Figure 2:**
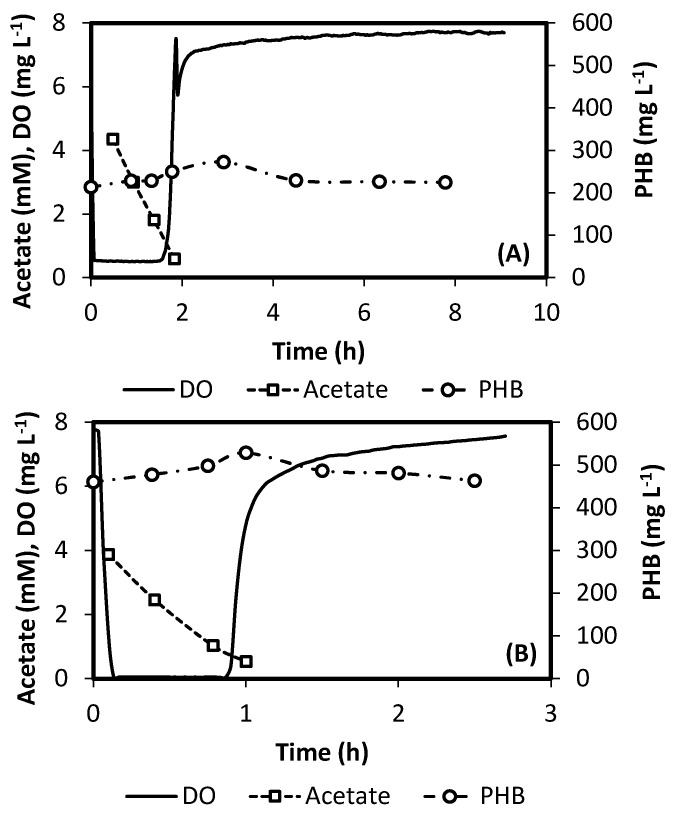
Evolution of the concentrations of acetate, DO and PHB during a typical operation cycle. (**A**) R0.2 (**B**) R0.6 SBR. In both cases feed concentration was 30 mM acetate.

**Figure 3:**
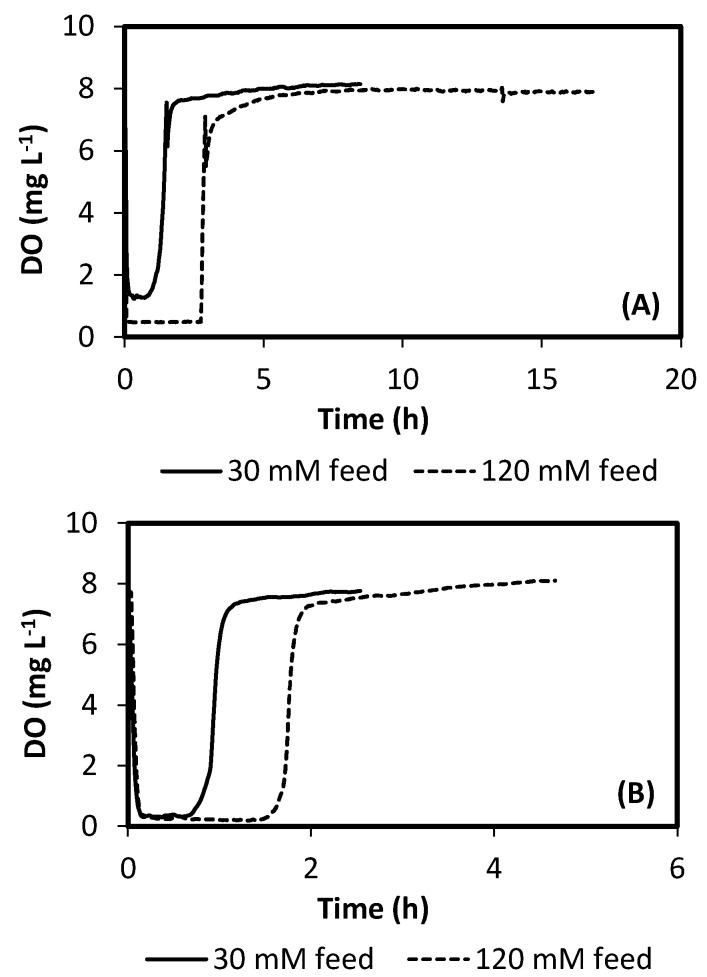
Evolution of DO concentration during operation of controlled SBRs. (**A**) R0.2, (**B**) R0.6.

Figure 2 presents typical operation cycles for R0.2 and R0.6. The dynamics of oxygen, acetate and PHB concentrations can be observed along a cycle with a feed of 30 mM acetate. Both cycles are quite different in total length, as a result of differences in applied F/F, by means of the DO-based control strategy. Operations of R0.2 and R0.6 were performed under fully automatic regulation of cycle time. The behavior of the F/F control can be further checked observing Figure 3. It represents typical DO evolutions during SBR cycles operated with 30 and 120 mM feeds, for reactors R0.2 and R0.6. Figure shows how the end of feast phase is characterized by a sudden increase in DO. Such behavior enables the use of a simple threshold value to determine the limit between feast and famine. During this research a value of 5 mg/L was used, which showed to be a good criterion, based on observation of DO profiles.

**Figure 4:**
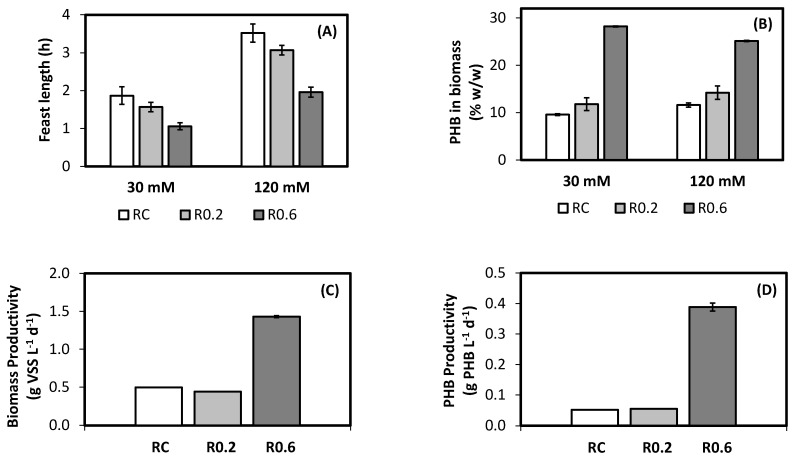
Observed behaviour of SBR at different operational conditions: (**A**) feast time, (**B**) minimum PHB biomass content (i.e., that at the beginning of feast cycle), (**C**) biomass productivity, (**D**) PHB productivity. Bars indicate standard deviation between replicas.

Figure 4A presents the feast length observed during each of the tested conditions. As expected, higher influent concentration produced longer feast lengths, since more time is needed for substrate consumption. During the operation of RC, total cycle time was fixed. This produced different F/F ratios at different influent concentrations, because of the variation of feast length. Average F/F ratios on RC were 0.19 for the 30 mM of feed concentration, and 0.42 for 120 mM. Average organic loading rate (OLR) for RC, considering both influent concentrations, was 1.1 g-acetate L^−1^ d^−1^. Cycle times for R0.2 and R0.6 were dependent on the influent concentration, which determined the feast length. Feast length and the applied F/F determined famine duration. In the case of R0.2, cycle times were 9 and 17 h, for influent concentrations 30 and 120 mM, respectively. Resulting average OLR was 0.9 g-acetate L^−1^ d^−1^. In the case of R0.6, cycle times were about 2.6 and 4.6 h, for influent concentrations 30 and 120 mM, respectively. Resulting average OLR was 3.4 g-acetate L^−1^ d^−1^. Shorter cycles mean more cycles per day, indicating a more frequent feed and therefore higher OLR. 

T-student tests (two-tailed, α = 0.05) showed that feast phase lengths were significantly shorter in the case of R0.6, at both 30 and 120 mM feed acetate concentration. This phenomenon would be the result of higher average substrate uptake rate, which could be related with the development of a more active biomass in the case of R0.6. This in turn may have resulted from the higher OLR applied on R0.6, because of shorter famine. These results are in agreement with Albuquerque et al. [13], who observed increases in acetate consumption rate as a result of an increment on OLR. 

Figure 4B presents biomass PHB content, determined at the beginning of the feast cycle. This corresponds to the minimum PHB content, since during feast PHB content of biomass increases, and during famines, it decreases (Figure 2). Then, maximum PHB content in the reactor is obtained close to the end of feast cycle. It has been shown that minimum and maximum PHB contents are related [18]. In the case of this research, during famine, in average, 20% of the PHB was consumed. The condition imposed in R0.6 derived into a higher accumulation of PHB in biomass. PHB contents observed for R0.2 and RC PHB were similar, not presenting statistical difference (*t*-test, two-tailed, α = 0.05). These results indicate that an increase in feed frequency (product of a shorter cycle time), would increase the storage response, as long as enough famine time exists to promote selection for PHB accumulating microorganisms. The fact that feed frequency would strongly affect the PHB production under F/F conditions has been widely corroborated [19,20,23]. Thus, it can be inferred that conditions imposed in R0.6 were positive for a better response in terms of PHB production, i.e., application of a high OLR, with a famine length that is long enough to promote biomass selection. 

The high feed frequency promoted in R0.6, and the concomitant high OLR, resulted in a biomass productivity that was about three times the one observed for RC and R0.2 (Figure 4C). When biomass productivity is combined with PHB content, PHB productivity can be evaluated, which is presented in Figure 4D. Differences between R0.6 and the other reactors are clearly augmented when performance is expressed as PHB productivity. This is the result of the effect that the higher fed frequency had on PHB content and biomass productivity, which multiplicate themselves when evaluating PHB productivity. This agrees with previous studies. For example, Valentino et al. [23] reported that when the cycle time is reduced by an increase in OLR, an increase in the polymer production yield can be observed, which can favor the productivity. Moreover, other studies have indicated that under non-regulated F/F ratios low substrate inputs could provoke time losses, which could affect the productive capacity for biological PHB production [19,24,25]. Then, it can be inferred that the application of the tested strategy is a simple and reliable way to promote a better performance of feast/famine-based bioreactors involving MMC for PHB production. 

### 3.2. Batch Experiments for PHB Production

Table 2 presents the results of the batch tests for PHB accumulation. As expected, the overall PHB contents reached under these experiments were higher than those observed in respective SBRs. In general terms, increases in PHB content were between 50 and 65% of the initial value. R0.6 generated the highest concentrations of PHB, as a result of an already high starting point. However, the maximum observed contents were lower than those observed in other studies, where PHB from batch experiments have reached values up to 80% of the dry weight [26,27]. Indeed, time required for consumption of acetate during these assays were higher than some of the accumulation experiments reported in literature [28,29]. Differences are probably related with the imposed conditions, especially substrate dosage strategy, since during this research acetate was fed only at the beginning of the assay. 

## 4. Discussion: Overall Assessment of Proposed Strategy

Integration of PHB producing processes with waste treatment facilities great opportunities for carbon recovery [30]. Biogas is a traditional way to valorize wastes through energy production. However, low market price of methane opens the possibility for the generation of high value-added products from organic wastes, such as PHAs [31]. Nevertheless, wastes are by definition variable in terms of concentration and composition. Most installations dedicated to waste treatment include large pre-treatment units, like primary settling tanks in sewage treatment, that can act as buffers. However, variations in flow and concentration can still be significant. As already commented, changes in operational conditions, such as feed concentration and organic load, can induce great changes in the performance of MMC for PHA production based on F/F operation strategy. This research proposes and validates a simple strategy for controlling SBRs using the evolution of dissolved oxygen concentration to effectively apply the desired F/F ratio, independent of changes in feed concentration. Implementation of DO-based control of F/F ratios requires simple instrumentation and control hardware. Moreover, it is based on the determination of threshold DO value, so its implementation requires very simple control routines. Results presented show that its application can successfully provide the desired F/F, despite the frequent changes in feed concentration, and therefore on OLR. Operation of R0.6 showed that control of F/F can be of great benefit for MMC for PHB production. Indeed, conditions enabled the operation at a larger load, preventing unnecessary famine periods that may result when operating under fixed pre-defined cycles, as was the case of RC.

## 5. Conclusions

A DO-based control strategy is presented and validated for the production of PHB, using SBRs operated using F/F cycles. Following DO evolution, system was able to identify the boundary between feast and famine, providing fixed relation between these operation stages, irrespective of variation of influent concentration. The reactor operated at F/F ratio of 0.6 promoted higher biomass productivities and PHB content, as a result of a better use of the time, preventing unnecessary famine times that will occur on a non-controlled system, when substrate concentration is lower than the expected one. This kind of control strategy is expected to be useful to maintain highly productive reactors, when wastes or waste-derived substrates are used for PHB production, because of natural changes in composition, concentration, or feed flow.

## Figures and Tables

**Table 1:** Operation conditions of SBR reactors used in this study.

Conditions	R0.2	R0.6	RC
F/F ratio	0.2	0.6	Variable, depending on substrate concentration
Cycle time	Variable, depending on substrate concentration	Variable, depending on substrate concentration	12 h
Influent acetate concentration	Alternating 30 and 120 mM	Alternating 30 and 120 mM	Alternating 30 and 120 mM

**Table 2:** Kinetics of batch PHB accumulation experiments.

Biomass Origin	Substrate Uptake Rate (g L^−1^ h^−1^)	Initial PHB Content (% *w*/*w*)	Final PHB Content (% *w*/*w*)
RC	0.04	7.0	11.6
R0.2	0.09	16.6	25.0
R0.6	0.09	29.8	44.5

## Data Availability

Not applicable.
